# Lithium battery supply chain and waste, and lithium and per- and polyfluoroalkyl substances in drinking water in the United States

**DOI:** 10.1016/j.ecoenv.2026.120991

**Published:** 2026-10-29

**Authors:** Shiwen Li, Eryn Uemori

**Affiliations:** Department of Public Health Sciences, Thompson School of Social Work and Public Health, University of Hawaiʻi at Mānoa, Honolulu, HI, USA

**Keywords:** PFAS, Lithium, Lithium-ion Battery, Drinking Water, United States

## Abstract

Rechargeable lithium-ion batteries (LiBs) are central to the clean energy transition, but their production, use, and disposal may introduce contaminants into drinking water systems. Using U.S. EPA Fifth Unregulated Contaminant Monitoring Rule (UCMR5) data, we evaluated whether ZIP codes with LiB waste activity or LiB facilities had higher lithium or per- and polyfluoroalkyl substance (PFAS) burdens in drinking water. LiB waste activity was identified from the 2023 EPA Biennial Hazardous Waste Report, and LiB facilities were identified from the NAATBatt Lithium-Ion Battery Supply Chain Database. Among 17,860 ZIP codes, 254 had LiB waste activity and 633 had at least one LiB facility. In adjusted conventional regression models, waste presence (β = −0.464, 95% CI: −0.919, −0.010) and facility presence (β = −0.491, 95% CI: −0.786, −0.196) were inversely associated with lithium concentrations, but these associations were attenuated and no longer statistically significant in spatial-error models. PFAS findings were mixed. Waste presence was not associated with summed PFAS concentration (β = −0.051, 95% CI: −0.175, 0.073), while shipped waste tonnage showed a borderline positive association (β = 0.259, 95% CI: 0.000, 0.518) that was attenuated after spatial adjustment. In spatial-error models, facility presence (β = 0.067, 95% CI: 0.015, 0.120) and facility count (β = 0.086, 95% CI: 0.026, 0.146) were positively associated with summed PFAS concentration. Overall, associations were sensitive to spatial dependence and varied across LiB indicators. These exploratory findings do not establish that LiB activities caused PFAS contamination but suggest that LiB facility locations warrant further investigation as indicators of industrial environments with elevated or more complex PFAS contamination.

## 1. Introduction

Rechargeable Lithium-ion batteries (LiBs) are the center of sustainable energy (Ngoy et al., 2025). They have been used in a wide range of electronic devices, including cell phones, laptops, and electric vehicles (EVs) that are important for daily life activities (Ngoy et al., 2025; Sciatti et al., 2026). Especially, EV adoption, which is the largest user of LiBs with a growing demand, (Wesselkaemper et al., 2025; Zhang et al., 2025) has shown environmental benefits such as air pollution reduction through the reduction of greenhouse gas release, (Bi et al., 2026; Eckel et al., 2026) and eventually leading to health benefits of potential reduction of asthma attacks and premature deaths related to air pollution (Zhu et al., 2023; Pennington et al., 2024).

Although these LiBs can be recycled, some studies suggest that only 5–10% of the LiBs are currently recycled globally, (Etude et al., 2024; Sederholm et al., 2025) and it is projected that there will be 8 million tons of LiB waste by 2040 (Baum et al., 2022). Improper disposal of LiBs can result in contamination of drinking water sources (Bolan et al., 2021). A previous study suggests that lithium contamination may be more prevalent than expected. For example, in groundwater, lithium was above the non-regulatory health-based screening level in 56% of the groundwater and 13% of the surface water samples (Lindsey et al., 2021).

It has been well established that at higher doses (therapeutic doses), lithium is used for treating mood disorders such as bipolar disorder through its potential role in the central nervous system (Alda, 2015; Sakrajda and Rybakowski, 2025). However, less is known about the health impact of the environmental level of lithium. Epidemiological studies have shown mixed findings on lithium in drinking water with adverse health impact, including dysregulated thyroid function, (Broberg et al., 2011) increased risk of Autism Spectrum Disorder, (Liew et al., 2023) but lower risk of dementia, (Kessing et al., 2017; Fraiha-Pegado et al., 2024) cancer, (Luo et al., 2025) and suicide rates (Memon et al., 2020). Due to the potential health effects of lithium in drinking water, the US Environmental Protection Agency (EPA) measured lithium in public drinking water systems in the Fifth Unregulated Contaminant Monitoring Rule (UCMR5) to improve understanding of the prevalence of contamination and potentially set new regulations (US EPA).

Besides lithium, per- and polyfluoroalkyl substances (PFAS), specifically bis-perfluoroalkyl sulfonimides (bis-FASIs), have been increasingly used to enhance the performance and stability of LiBs. One recent paper suggested that bis-FASIs are released to the soil, sediment, and surface water around the manufacturing areas of LiBs, and bis-FASIs are as toxic as the other legacy PFAS, such as perfluorooctanoic acid (PFOA) and perfluorooctane sulfonate (PFOS) (Guelfo et al., 2024). However, bis-FASIs were not included among the PFAS monitored under UCMR5. Thus, the UCMR5 PFAS data used in this study provide a broader measure of PFAS occurrence rather than a direct measure of battery-specific PFAS contamination.

Given the opportunity of measures that both PFAS and lithium were measured in UCMR5, in the present study, we evaluated whether zone improvement plan (ZIP) codes with LiB waste activity also show higher lithium or PFAS burden in drinking water and whether a broader battery facility footprint exhibits a similar pattern.

## 2. Materials and methods

### 2.1. PFAS and lithium in drinking water

Drinking-water data were obtained from U.S. EPA Fifth Unregulated Contaminant Monitoring Rule (UCMR5) records, which were conducted between 2023 and 2025. We linked drinking-water data to ZIP codes through a public water system (PWS)-ZIP code crosswalk provided by UCMR5.

PWSs report the ZIP codes they serve directly to the EPA as part of the mandatory data elements submitted during the monitoring cycle. Therefore, one ZIP code can be served by multiple water systems, and one water system can serve many ZIP codes. However, PWS-ZIP crosswalk should be interpreted as an approximate geographic linkage rather than a precise assignment of water service to all households within a ZIP code.

PFAS measures were derived from ZIP-code level analyte-specific summaries collapsed into summed PFAS concentration, any PFAS detection, and the number of detected PFAS analytes. Lithium measures included ZIP-code level mean.

For each PWS and PFAS analyte, we calculated the mean of all available UCMR5 monitoring results. We linked these PWS-specific analyte means to all ZIP codes reported as served by each PWS. For ZIP codes linked to multiple PWSs, we calculated the unweighted mean of the PWS-specific means for each analyte. The summed PFAS concentration was then calculated as the sum of these ZIP-level analyte-specific mean concentrations across all PFAS analytes.

UCMR5 monitored 29 PFAS. bis-FASIs, which have been identified in prior studies of LiB components, were not among the monitored analytes. Accordingly, the summed PFAS measure represents aggregate concentrations of UCMR5-monitored PFAS and should not be interpreted as a measure of battery-specific PFAS contamination. We additionally summarized the relative contribution of individual PFAS analytes to the aggregate summed PFAS concentration.

### 2.2. LiB waste and facility

The main exposure, LiB waste activity, was constructed from the 2023 EPA Biennial Hazardous Waste Report (US EPA). The Biennial Hazardous Waste Report is a federal reporting system under the Resource Conservation and Recovery Act that compiles information on the generation, management, and disposition of hazardous waste in the US. It is primarily submitted by large quantity generators, and includes facility-level information on hazardous waste types, quantities generated, and whether wastes were treated, stored, disposed of, recycled, or shipped off-site.

We identified LiB waste records focusing on “lithium-ion batteries” and “lithium-metal batteries”, including damaged batteries and batteries contained in or packed with equipment, while excluding non-battery lithium chemical wastes such as electrolyte-only descriptions. ZIP-code level waste measures included any LiB waste activity, shipped tons, and receiver counts.

We obtained LiB facility data from the National Alliance for Advanced Technology Batteries (NAATBatt) Lithium-Ion Battery Supply Chain Database, (NLR and NAATBatt International, n.d) a curated North American database developed by NAATBatt International and NREL that identifies companies and facilities involved in the LiB supply chain. The database includes upstream, midstream, and downstream segments, ranging from raw materials and battery materials processing to cell and pack manufacturing, recycling, and reuse, and provides facility-level information on location, product/facility type, supply-chain segment, production capacity, and project status.

For our analysis, we summarized LiB facilities at the ZIP-code level to create a binary presence variable and a facility count variable.

### 2.3. Covariates

Covariates were obtained from the 2023 American Community Survey and from the 2020 USDA, Economic Research Service’s (ERS) Rural-Urban Commuting Area (RUCA) codes at the ZIP code level (Rural-Urban, n.d). We included the following covariates: state, population, median household income, poverty, educational attainment, racial and ethnic composition, age structure, RUCA category, and number of PWSs within a ZIP code. Population density based on the 2020 Census Bureau at ZIP Code Tabulation Area (ZCTA) level was also included.

### 2.4. Statistical analysis

We used multivariable linear regression to evaluate associations of LiB waste activity and LiB facility measures, modeled separately, with lithium and summed PFAS concentrations in drinking water. Because lithium and summed PFAS concentrations were right-skewed, both outcomes were modeled using a log(1 + x) transformation. Continuous exposure measures, including waste shipped tons and facility count, were also modeled using a log(1 + x) transformation.

To improve interpretability, coefficients from models with log(1 + concentration)-transformed outcomes were back-transformed. For binary exposure indicators, 100 × [exp(β)–1] represents the approximate percentage difference in contaminant concentration between exposed and unexposed ZIP codes. For continuous exposures that were also modeled using log(1 + x), we report the percentage difference associated with a doubling of exposure + 1, calculated as 100 × [exp(βln2) – 1] or 100 × [2^β^ – 1].

For the number of detected PFAS analytes, we used quasi-Poisson regression due to overdispersion. Primary models adjusted for state, population size, median household income, poverty, educational attainment, racial and ethnic composition, age structure, number of linked public water systems, population density, and RUCA category. In sensitivity analyses, LiB waste models additionally adjusted for battery facility count, and LiB facility models additionally adjusted for hazardous-waste receiver count.

We further conducted exploratory analyses stratified by U.S. region (Northeast, Southeast, Midwest, Southwest, and West). Because the numbers of ZIP codes with LiB waste activity or facilities were limited within some regions, these analyses were intended to assess possible geographic variation in the direction and magnitude of associations rather than provide definitive region-specific estimates.

Because drinking-water contaminant concentrations exhibited geographic clustering, we evaluated residual spatial autocorrelation and conducted spatial regression sensitivity analyses. We constructed an 8-nearest-neighbor spatial weights matrix based on representative points within ZCTAs. Moran’s I was used to assess spatial autocorrelation in residuals from the adjusted linear regression models. We then refit the continuous lithium and summed PFAS models using generalized method-of-moments spatial-error regression with row-standardized spatial weights and the same covariates as the primary models. Spatial-error models account for residual spatial dependence through an autoregressive error parameter, λ.

## 3. Results

Among 17,860 ZIP codes included in the analysis, 254 had LiB waste activity and 633 had at least one LiB facility (Table 1). ZIP codes with LiB waste activity had slightly higher mean lithium concentrations than ZIP codes without waste activity. Although mean summed PFAS concentrations were similar between ZIP codes with and without LiB waste activity (4.75 vs. 4.81 ng/L), waste-active ZIP codes had a higher median summed PFAS concentration (0.64 vs. 0.00 ng/L), a higher median number of detected PFAS analytes (1 vs. 0), and a higher prevalence of any PFAS detection (61.8% vs. 47.6%). The large differences between the means and medians, together with SDs substantially exceeding the means, indicate strongly right-skewed PFAS concentration distributions. Thus, the higher frequency and number of PFAS detections in waste-active ZIP codes did not correspond to a higher arithmetic mean summed PFAS concentration, which may partly reflect the influence of high-concentration observations in the upper tails of the distributions. ZIP codes with LiB facilities had lower mean lithium concentrations but higher mean and median summed PFAS concentrations, more detected PFAS analytes, and a higher prevalence of PFAS detection than ZIP codes without facilities. An analyte-specific descriptive analysis showed that the summed PFAS concentration was primarily driven by PFBA (20.85%), PFPeA (16.64%), PFOS (13.17%), PFHxA (12.29%), PFBS (11.88%), and PFOA (10.99%), which together accounted for 85.81% of the aggregate summed PFAS concentration (Supplemental Table 1).

ZIP codes with LiB-related activity also differed substantially in several demographic and geographic characteristics. Waste-active ZIP codes had larger populations than ZIP codes without waste activity (34,003 vs. 16,829), were more frequently metropolitan-core areas (84.6% vs. 49.0%), and had higher population density, a greater Hispanic population proportion, and fewer adults aged 65 years or older. Similar patterns were observed for LiB facility ZIP codes, which had larger populations (31,192 vs. 16,555), higher median household income ($93,338 vs. $79,509), a greater proportion of residents with a bachelor’s degree or higher (0.42 vs. 0.30), higher population density, and were more frequently metropolitan-core areas (83.6% vs. 48.3%). These descriptive differences indicate that LiB waste activity and facilities were concentrated predominantly in more populated and urban ZIP codes.

Spatially, PFAS and lithium showed distinct geographic patterns (Fig. 1). PFAS concentrations appeared elevated in selected areas of the Southeast, southern California, Texas, and parts of the Midwest and Northeast, while lithium concentrations were more pronounced in parts of the Mountain West, Southwest, and central US. Panel C provides a composite visualization of elevated lithium and summed PFAS concentrations together with LiB waste activity and facility locations, facilitating comparison of spatial co-location patterns.

In adjusted models, lithium battery waste activity was inversely associated with lithium mean concentration (β = −0.464, 95% CI: −0.919, −0.010), corresponding to an approximate 37.1% lower lithium concentration in ZIP codes with versus without waste activity (95% CI: 60.1–1.0% lower). A doubling of shipped tons was associated with an approximate 51.1% lower lithium concentration (β=−1.033, 95% CI: −1.981, −0.085; percentage difference: 74.7% to 5.7%). (Fig. 2). Battery facility presence was associated with an approximate 38.8% lower lithium concentration (β=−0.491, 95% CI: −0.786, −0.196; percentage difference: −54.4% to −17.8%), while a doubling of facility count was associated with an approximate 29.1% lower lithium concentration (β=−0.497, 95% CI: −0.830, −0.164; percentage difference: −43.7% to −10.7%).

Associations with summed PFAS concentration were weaker. Waste presence was not clearly associated with summed PFAS concentration (β=−0.051, 95% CI: 0.175, 0.073), corresponding to an approximate 5.0% lower concentration, with a confidence interval ranging from 16.1% lower to 7.6% higher. A doubling of shipped tons corresponded to an estimated 19.7% higher summed PFAS concentration (β=0.259, 95% CI: 0.000, 0.518; approximate percentage difference: 0.0–43.2%), but this estimate was borderline and should be interpreted cautiously. Facility presence corresponded to an estimated 6.7% higher summed PFAS concentration (β=0.065, 95% CI: −0.016, 0.146; percentage difference: −1.6–15.7%), and a doubling of facility count corresponded to an estimated 4.5% higher concentration (β=0.063, 95% CI: −0.028, 0.154; percentage difference: −1.9–11.3%). The confidence intervals for both facility measures included the null.

Sensitivity analyses that adjusted for waste or facility count in each other’s models showed similar results but attenuated statistical significance. Full summary statistics are available in Supplemental Table 2.

In exploratory region-stratified analyses (Fig. 3), precision was limited by the relatively small numbers of ZIP codes with LiB-related activity within individual regions. Waste-active ZIP codes numbered 32 in the Northeast, 59 in the Southeast, 61 in the Midwest, 29 in the Southwest, and 68 in the West. The corresponding numbers of LiB facilities were 134, 203, 225, 92, and 249, respectively; ZIP codes containing at least one LiB facility numbered 97, 154, 144, 74, and 162 (Supplemental Table 3). Only 5 of the 40 region-specific associations shown in Fig. 3A had p < 0.05, and several confidence intervals were wide. Accordingly, these findings should be regarded as exploratory and should not be interpreted as definitive evidence of regional heterogeneity. Full regional model estimates are provided in Supplemental Table 4.

Shown in Supplemental Table 5, Moran’s I for residuals from the adjusted models was approximately 0.54 for lithium and 0.57 for summed PFAS models (all p < 0.001). Spatial-error parameters were similarly large (λ=0.82, p < 0.001), indicating substantial residual geographic dependence after adjustment for measured covariates. Accounting for this dependence attenuated the inverse lithium associations observed in the primary models. Waste presence (β=−0.202, 95% CI: −0.505, 0.102), shipped waste (β=−0.136, 95% CI: −0.761, 0.488), facility presence (β=−0.138, 95% CI: −0.336, 0.060), and facility count (β=−0.096, 95% CI: −0.320, 0.129) were no longer statistically associated with lithium concentration.

Waste-related PFAS associations were also attenuated. In particular, the borderline positive association with shipped waste observed in the primary model was substantially reduced in the spatial-error model (β=0.057, 95% CI: −0.109, 0.224; p = 0.499), corresponding to an approximate 4.1% difference per doubling of shipped tons. In contrast, facility presence remained positively associated with summed PFAS in the spatial model (β=0.067, 95% CI: 0.015, 0.120; p = 0.012), corresponding to approximately 7.0% higher summed PFAS concentration. A doubling of facility count was associated with approximately 6.2% higher summed PFAS (β=0.086, 95% CI: 0.026, 0.146; p = 0.005).

## 4. Discussion

In the present study, we evaluated whether ZIP-code-level LiB waste activity and facility indicators were associated with lithium and summed PFAS concentrations in drinking water. PFAS findings were inconsistent across exposure indicators and generally imprecise. Waste presence was not associated with higher summed PFAS concentration, while shipped waste tonnage showed only a borderline positive association. Facility presence and facility count showed small positive estimates, but the confidence intervals included the null in the primary models. These findings should therefore be considered suggestive and exploratory rather than evidence that LiB activities contributed to PFAS contamination. The conventional regression models showed inverse associations between LiB waste and facility indicators and lithium concentrations, although these associations were sensitive to adjustment for spatial dependence.

The demographic differences observed in Table 1 also highlight the importance of accounting for characteristics associated with the location of LiB-related activities. ZIP codes with LiB waste activity or facilities were generally more populous, more densely populated, and more urban than ZIP codes without these activities, and they also differed in socioeconomic, racial and ethnic, and age composition. These characteristics may be associated both with the siting of industrial activities and with drinking-water contaminant patterns through differences in source waters, infrastructure, land use, and other co-occurring industrial activities (Mohai and Saha, 2015; Hu et al., 2016; McMahon et al., 2022). We therefore adjusted the primary models for population size, median household income, poverty, educational attainment, racial and ethnic composition, age structure, population density, RUCA category, and the number of linked public water systems. Nevertheless, residual confounding by unmeasured characteristics of urban and industrial environments cannot be excluded.

Substantial residual spatial autocorrelation was present, and these associations were attenuated to the null in spatial-error sensitivity analyses. These findings indicate that the inverse lithium associations and the borderline waste-PFAS association observed in conventional models were sensitive to geographic dependence and should therefore be interpreted cautiously. However, positive associations of LiB facility presence and facility count with summed PFAS concentration persisted and became more apparent after accounting for spatial dependence.

The direction of some PFAS point estimates is aligned with emerging evidence that PFAS are used in LiB components and may be released during manufacturing, use, or disposal. However, our ecological analysis could not identify specific PFAS sources or establish that LiB-related activities caused the observed drinking-water concentrations. For example, Guelfo et al. found that LiB components use PFAS that can be released during manufacture, use, and disposal (Guelfo et al., 2024). Similar findings were reported in studies in China. In one study, short-chain PFAS were detected in both wastewater and receiving waters in the LiB industrial area. In another study, at least 9 PFAS were detected in each of the environmental samples, including soil, dust, water, and sediment near a LiB recycling area (Qi et al., 2024). These prior studies suggest that some stages of LiB production, recycling, or disposal may represent potential environmental sources of PFAS. PFAS have been linked to a wide range of adverse health outcomes, including low birth weight, high cholesterol, liver damage, and several cancers (Fenton et al., 2021). With the growing demand for EVs, localized supply chains for LiB in the U.S. may be economically beneficial for the companies and residents in the nearby towns, but the potential health risk should not be ignored.

Importantly, UCMR5 did not monitor the bis-FASIs highlighted in prior studies of LiB components, and the summed PFAS outcome in the present study combined PFAS that are not specific to LiB-related sources. Analyte-specific analyses showed that the summed concentration was predominantly driven by PFBA, PFPeA, PFOS, PFHxA, PFBS, and PFOA, which together accounted for approximately 86% of the aggregate summed PFAS concentration. These compounds may originate from multiple environmental and industrial sources. Consequently, the observed associations cannot determine whether the measured PFAS originated from LiB-related activities and may instead reflect broader industrial PFAS contamination or spatial co-location.

The inverse lithium associations were less expected and likely reflect a different process of distribution of lithium in drinking water that is not driven by LiB facilities or waste. The substantial attenuation of the lithium associations in spatial-error models suggests that the inverse associations observed in conventional regression were driven, at least in part, by broad geographic structure not fully captured by measured ZIP-code characteristics and state adjustment. Geographic variation in source-water geology, aquifer mineral composition, hydrochemistry, and other spatially patterned characteristics may contribute to this residual dependence. Lithium concentrations in groundwater can vary substantially across aquifer types because lithium is released through weathering and dissolution of lithium-bearing minerals and may be further mobilized through water–rock interaction and cation-exchange processes (Lindsey et al., 2021). Lithium concentrations in drinking water may be governed more by source-water geology, aquifer mineral composition, and regional hydrochemistry than by ZIP code-level battery waste or facility measures (Lindsey et al., 2021; Guo et al., 2026). Groundwater age, evaporative concentration in arid environments, and mixing with geothermal fluids, saline groundwater, or brines may further increase lithium concentrations in some regions (Lindsey et al., 2021; Guo et al., 2026). Because conventional drinking-water treatment may have limited effectiveness for removing dissolved lithium, geographic differences in source-water hydrochemistry can persist into finished drinking water (Guo et al., 2026). The pattern is visually clear, and based on Fig. 1, we can observe the strong spatial clustering of lithium contamination. Exploratory region-stratified analyses suggested geographic variation in the point estimates. However, the number of exposed ZIP codes was limited within several regions, and confidence intervals were wide. Only 5 of the 40 region-specific estimates presented in Fig. 3A were nominally statistically significant. These results therefore provide only preliminary evidence of possible regional differences and should not be interpreted as demonstrating effect heterogeneity. Larger studies with more geographically resolved exposure information are needed to evaluate whether associations truly differ across regions. The inverse association between LiB facility count and lithium was strongest in the Midwest and Southeast. PFAS estimates varied across regions and were generally imprecise. The largest positive estimate for shipped waste was observed in the Southeast, but the region-stratified findings were exploratory and should not be interpreted as definitive evidence of regional effect heterogeneity. Another explanation is that battery facilities and waste sites may be located in urban-industrial ZIP codes whose served public water systems draw from sources with relatively low background lithium. Thus, battery-related activity may be a poor proxy for lithium in finished drinking water, even if it is environmentally relevant for other contaminants. In addition, the inverse lithium associations should not be interpreted as evidence that LiB facilities or waste activity reduce lithium concentrations in drinking water.

This study has several limitations. It was ecological and ZIP code-based, so it cannot identify individual exposure pathways or establish causality for individual-level exposure. Public water system-linked contaminant summaries may not correspond perfectly to household tap exposures because service areas and ZIP code boundaries differ. A PWS may serve only part of a ZIP code, while different portions of the same ZIP may be served by different systems with different contaminant concentrations. Conversely, one PWS may serve several ZIP codes, resulting in the same monitoring information contributing to multiple ZIP-level summaries and potentially inducing correlation among observations. This boundary mismatch may cause spatial misclassification. Therefore, the ZIP-level contaminant measures should be interpreted as area-level screening indicators rather than estimates of household-specific exposure. Lastly, hazardous waste and facility databases may incompletely capture lithium battery-related activity, especially historically.

In conclusion, substantial spatial dependence was present in drinking-water lithium and PFAS concentrations. The PFAS findings were inconclusive. Several point estimates were positive, but most confidence intervals included the null, and the positive estimate for shipped waste was only borderline. LiB-related activity may warrant further evaluation as a potential screening indicator, but the present results do not establish that ZIP codes with LiB waste or facilities have higher PFAS contamination. Further studies using finer spatial linkage, source-specific PFAS measurements, and information on relevant release pathways are needed. After accounting for this dependence, the inverse lithium associations and the borderline association between shipped LiB waste and PFAS observed in conventional models were attenuated and no longer statistically significant. In contrast, LiB facility presence and facility count were positively associated with summed PFAS concentrations in spatial-error models. These findings suggest that LiB facility locations may warrant further investigation as potential indicators of industrial environments with elevated PFAS contamination, while providing little evidence that LiB waste activity itself is associated with PFAS concentrations. Finer spatial linkage, source-specific measurements, and longitudinal data are needed to clarify these relationships.

## Supplementary Material

Supplementary material

## Figures and Tables

**Fig. 1.**
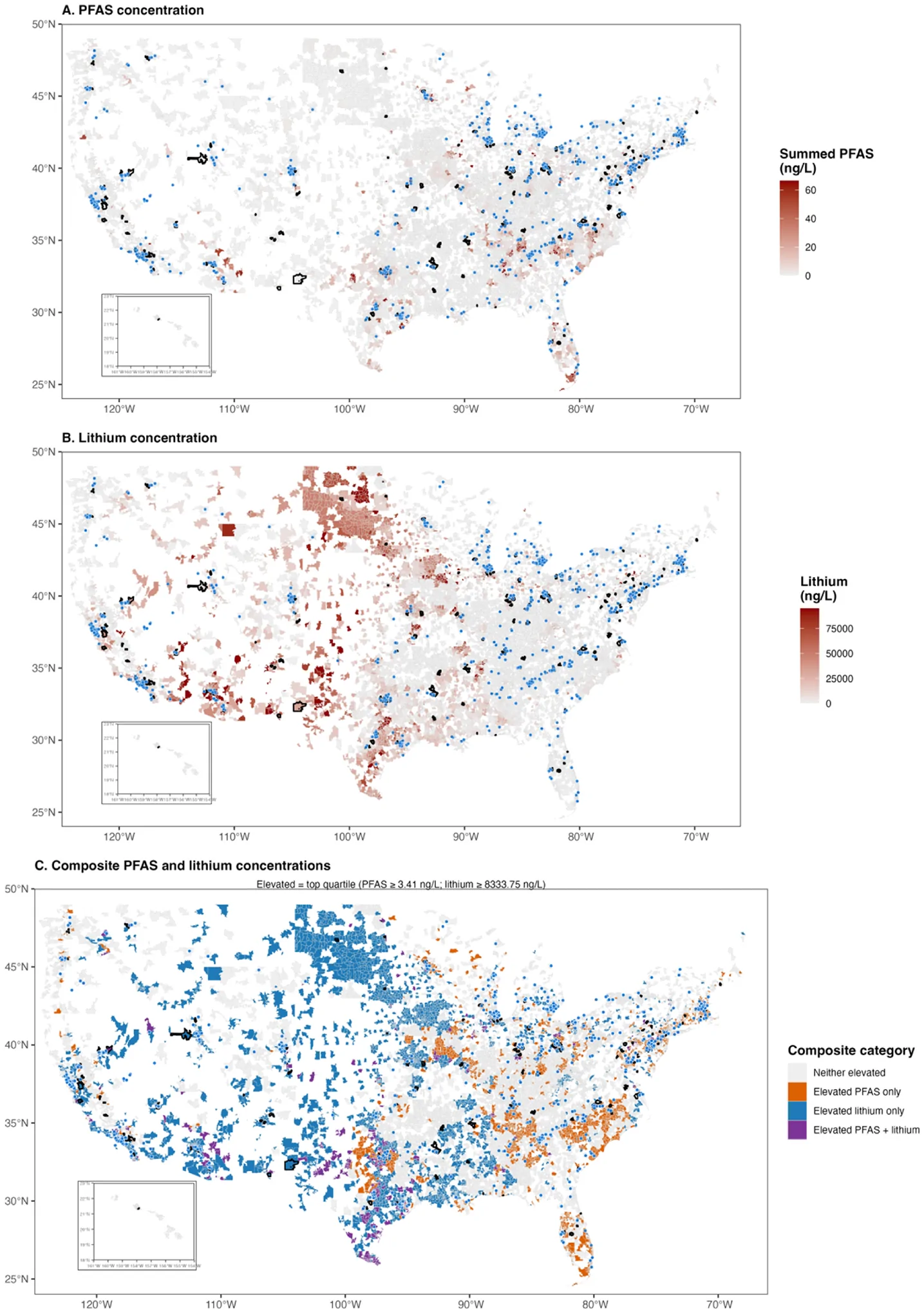
ZIP-code-level (A) summed PFAS concentrations, (B) lithium concentrations, and (C) composite spatial distribution of elevated summed PFAS and lithium concentrations, with LiB waste-active ZIP codes and facilities overlaid. * In panel C, elevated concentrations were defined as values in the highest quartile of the respective ZIP-code distributions. The composite panel is descriptive and does not represent an inferential analysis.

**Fig. 2.**
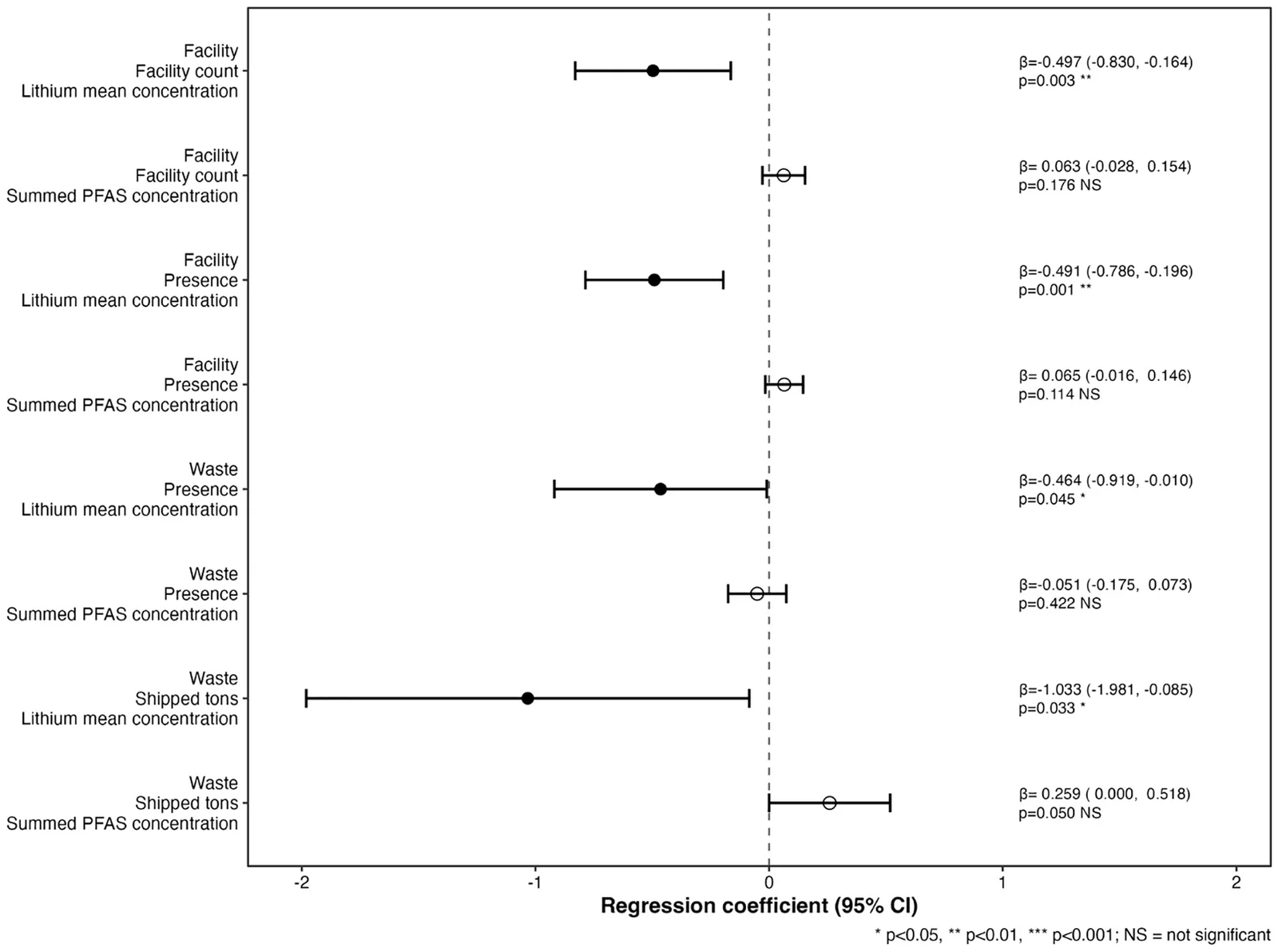
Adjusted associations of lithium-ion battery (LiB) waste activity and facility measures with lithium and PFAS concentrations. * Linear regression coefficients are from models of log(1 +contaminant concentration). For binary exposures, approximate percentage differences were calculated as 100 × [exp(β)–1]. For continuous log(1 +x)-transformed exposures, percentage differences per doubling of exposure + 1 were calculated as 100 × [2^β^–1].

**Fig. 3.**
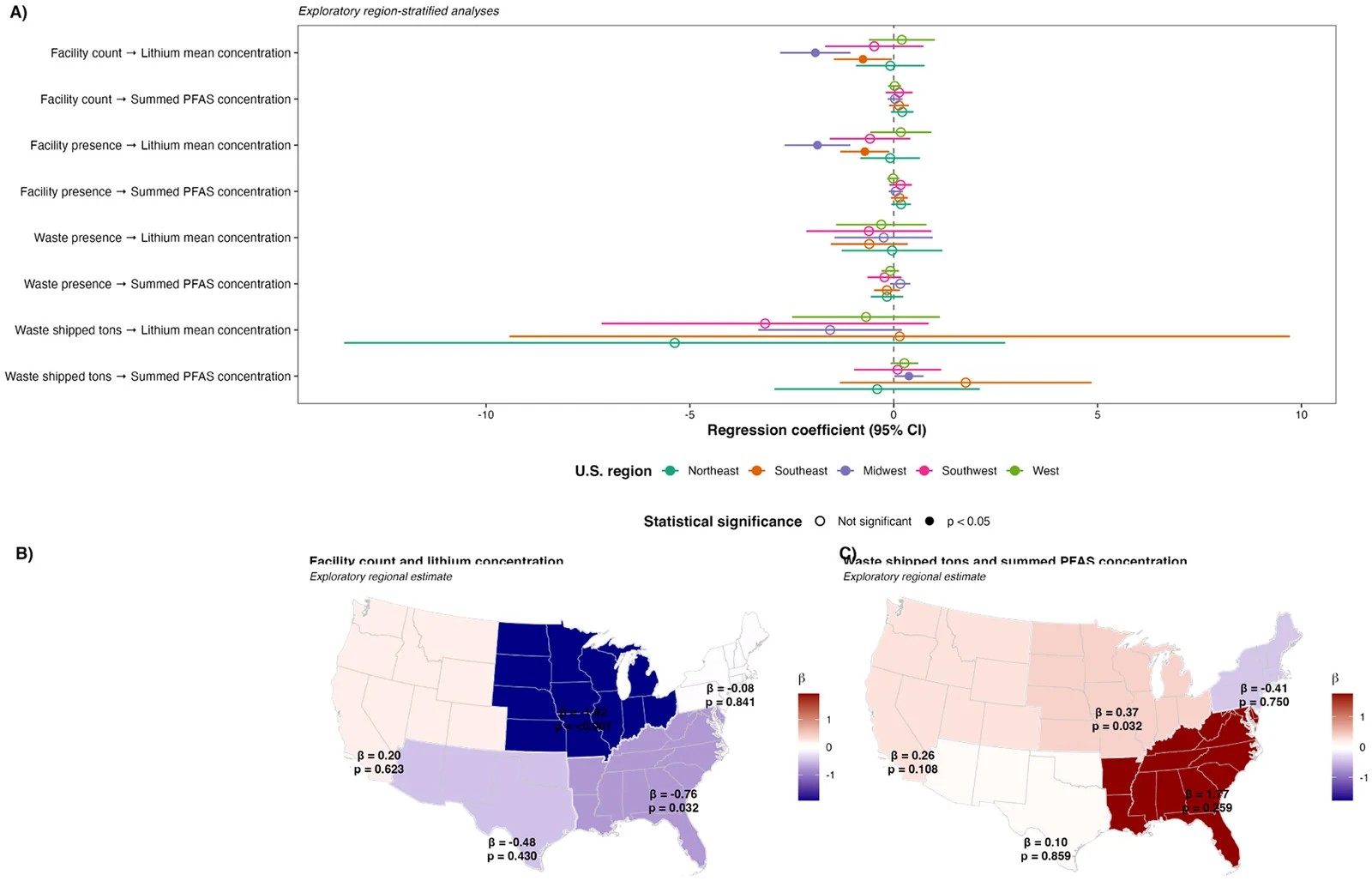
Stratified analysis of associations of lithium-ion battery (LiB) waste activity and facility measures with lithium and PFAS concentrations by US region. (A) Region-stratified associations between LiB facility/waste indicators and lithium or PFAS concentrations. (B) Regional beta coefficients for the association between LiB facility count and lithium concentration. (C) Regional beta coefficients for the association between LiB waste shipped tons and summed PFAS concentration. * Regional analyses were exploratory because stratification substantially reduced the numbers of ZIP codes with LiB waste activity or facilities; only 5 of 40 region-specific estimates in panel A had p < 0.05, and several confidence intervals were wide.

**Table 1:** ZIP-code characteristics by lithium-ion battery (LiB) waste activity and battery facility presence.

Characteristic	LiB Waste: No (N = 17,606)	LiB Waste: Yes (N = 254)	LiB Facility: No (N = 17,227)	LiB Facility: Yes (N = 633)
Lithium, ng/L				
Mean (SD)	8984.30 (21,995.34)	9626.72 (25,645.03)	9058.95 (22,039.85)	7210.70 (22,289.84)
Median (Q1, Q3)	0.00 (0.00, 8393.75)	0.00 (0.00, 7703.13)	0.00 (0.00, 8683.33)	0.00 (0.00, 3920.00)
Summed PFAS, ng/L				
Mean (SD)	4.81 (17.75)	4.75 (11.36)	4.77 (17.80)	5.75 (13.66)
Median (Q1, Q3)	0.00 (0.00, 3.48)	0.64 (0.00, 4.25)	0.00 (0.00, 3.41)	0.78 (0.00, 5.49)
Detected PFAS analytes				
Mean (SD)	1.86 (2.74)	2.39 (2.82)	1.84 (2.73)	2.64 (3.05)
Median (Q1, Q3)	0.00 (0.00, 3.00)	1.00 (0.00, 4.00)	0.00 (0.00, 3.00)	1.00 (0.00, 5.00)
Lithium detected				
No	10,505 (59.7%)	147 (57.9%)	10,246 (59.5%)	406 (64.1%)
Yes	7101 (40.3%)	107 (42.1%)	6981 (40.5%)	227 (35.9%)
PFAS detected				
No	9223 (52.4%)	97 (38.2%)	9061 (52.6%)	259 (40.9%)
Yes	8383 (47.6%)	157 (61.8%)	8166 (47.4%)	374 (59.1%)
Population	16,829.09 (17,159.20)	34,002.56 (20,345.53)	16,554.55 (17,088.10)	31,191.76 (17,846.89)
Median household income	79,918.60 (34,801.41)	85,568.88 (31,522.99)	79,508.82 (34,519.16)	93,337.92 (38,539.44)
Poverty proportion	0.13 (0.09)	0.13 (0.09)	0.13 (0.09)	0.12 (0.09)
Bachelor’s degree or higher	0.31 (0.18)	0.32 (0.16)	0.30 (0.18)	0.42 (0.20)
Non-Hispanic Black proportion	0.10 (0.16)	0.15 (0.19)	0.10 (0.17)	0.11 (0.16)
Hispanic proportion	0.13 (0.18)	0.25 (0.24)	0.13 (0.18)	0.16 (0.17)
Non-Hispanic Asian proportion	0.03 (0.07)	0.07 (0.11)	0.03 (0.07)	0.09 (0.12)
Population under 18	0.22 (0.06)	0.23 (0.05)	0.22 (0.06)	0.21 (0.06)
Population 65 or older	0.19 (0.08)	0.15 (0.05)	0.19 (0.08)	0.16 (0.05)
Linked public water systems	1.57 (0.98)	1.91 (1.13)	1.56 (0.97)	1.85 (1.19)
Population density, per km^2^	788.65 (2436.92)	1107.89 (3049.78)	776.78 (2444.73)	1239.55 (2465.42)
RUCA category				
Metropolitan core	8628 (49.0%)	215 (84.6%)	8314 (48.3%)	529 (83.6%)
Metropolitan	3127 (17.8%)	22 (8.7%)	3110 (18.1%)	39 (6.2%)
Micropolitan	2258 (12.8%)	15 (5.9%)	2232 (13.0%)	41 (6.5%)
Small town / Rural	3593 (20.4%)	2 (0.8%)	3571 (20.7%)	24 (3.8%)
Other	0 (0.0%)	0 (0.0%)	0 (0.0%)	0 (0.0%)

1 Continuous variables are presented as mean (SD). Lithium concentration, summed PFAS concentration, and detected PFAS analyte count are additionally presented as median (Q1, Q3). Categorical variables are presented as n (%).

## Data Availability

I have linked the data in the manuscript

## Appendix A. Supporting information

Supplementary data associated with this article can be found in the online version at doi:10.1016/j.ecoenv.2026.120991.

## Abbreviations

bis-FASIs: bis-perfluoroalkyl sulfonimides
EPA: Environmental Protection Agency
ERS: Economic Research Service
EV: electric vehicle
LiB: lithium-ion battery
NAATBatt: National Alliance for Advanced Technology Batteries
NREL: National Renewable Energy Laboratory
PFAS: per- and polyfluoroalkyl substances
PFOA: perfluorooctanoic acid
PFOS: perfluorooctane sulfonate
PWS: public water system
RUCA: Rural-Urban Commuting Area
UCMR5: Fifth Unregulated Contaminant Monitoring Rule
US: United States
ZCTA: ZIP Code Tabulation Area
ZIP: Zone Improvement Plan
