# Supplementary material for: Lithium battery supply chain and waste, and lithium and per- and polyfluoroalkyl substances in drinking water in the United States
Source: Ecotoxicol Environ Saf. Author manuscript; Available in PMC 2026 Nov 4. (PMC13728548; doi:10.1016/j.ecoenv.2026.120991)
Supplement: Supplementary file 1 [file NIHMS2220393-supplement-Supplementary_material.docx]

**Supplemental Materials**

**Title:** Lithium Battery Supply Chain and Waste, and Lithium and Per- and Polyfluoroalkyl Substances in Drinking Water in the United States

**Authors:** Shiwen Li^1*^, Eryn Uemori^1^

**Affiliations**:

1. Department of Public Health Sciences, Thompson School of Social Work and Public Health, University of Hawaiʻi at Mānoa, Honolulu, HI, USA

**Corresponding Author:**

Shiwen Li, PhD

Email: shiwenli@hawaii.edu

Phone: (808) 956-4308

Address: 1960 East-West Road, D104H, Honolulu, HI 96822

| Supplemental Table 1. Individual UCMR5 PFAS analytes contributing to the summed PFAS concentration in analytic ZIP codes. | | | | |
| --- | --- | --- | --- | --- |
| PFAS analyte | **ZIP codes with detection, n (%)** | **Mean concentration, ng/L** | **Contribution to aggregate summed PFAS, %** | **Cumulative contribution, %** |
| PFBA | 5,428 (30.4%) | 1.002 | 20.85 | 20.85 |
| PFPeA | 5,377 (30.1%) | 0.800 | 16.64 | 37.49 |
| PFOS | 3,610 (20.2%) | 0.633 | 13.17 | 50.66 |
| PFHxA | 4,557 (25.5%) | 0.591 | 12.29 | 62.94 |
| PFBS | 4,453 (24.9%) | 0.571 | 11.88 | 74.82 |
| PFOA | 3,476 (19.5%) | 0.528 | 10.99 | 85.81 |
| PFHxS | 2,681 (15.0%) | 0.267 | 5.56 | 91.37 |
| 6:2 FTS | 821 (4.6%) | 0.198 | 4.11 | 95.48 |
| PFHpA | 1,847 (10.3%) | 0.127 | 2.63 | 98.11 |
| HFPO-DA | 184 (1.0%) | 0.044 | 0.91 | 99.03 |
| PFNA | 503 (2.8%) | 0.026 | 0.53 | 99.56 |
| PFPeS | 172 (1.0%) | 0.009 | 0.19 | 99.75 |
| 8:2 FTS | 76 (0.4%) | 0.008 | 0.16 | 99.91 |
| PFDA | 31 (0.2%) | 0.001 | 0.03 | 99.94 |
| NFDHA | 9 (0.1%) | 0.001 | 0.02 | 99.96 |
| NMeFOSAA | 3 (<0.1%) | <0.001 | 0.01 | 99.97 |
| PFUnA | 34 (0.2%) | <0.001 | 0.01 | 99.98 |
| ADONA | 9 (0.1%) | <0.001 | 0.01 | 99.99 |
| PFHpS | 10 (0.1%) | <0.001 | <0.01 | 99.99 |
| PFDoA | 4 (<0.1%) | <0.001 | <0.01 | 99.99 |
| 4:2 FTS | 15 (0.1%) | <0.001 | <0.01 | 100.00 |
| PFMPA | 2 (<0.1%) | <0.001 | <0.01 | 100.00 |
| 9Cl-PF3ONS | 1 (<0.1%) | <0.001 | <0.01 | 100.00 |
| NEtFOSAA | 1 (<0.1%) | <0.001 | <0.01 | 100.00 |
| PFMBA | 1 (<0.1%) | <0.001 | <0.01 | 100.00 |
| 11Cl-PF3OUdS | 0 (0.0%) | 0 | 0.00 | 100.00 |
| PFEESA | 0 (0.0%) | 0 | 0.00 | 100.00 |
| PFTA | 0 (0.0%) | 0 | 0.00 | 100.00 |
| PFTrDA | 0 (0.0%) | 0 | 0.00 | 100.00 |

| Supplemental Table 2. Full summary statistics for the associations between lithium-ion battery waste and facility and PFAS and lithium in drinking water for both primary and sensitivity analysis. | | | | | | |
| --- | --- | --- | --- | --- | --- | --- |
| Lithium-ion Battery | **Exposure** | **Outcome** | **Estimate type** | **Result** | **P value** | **Analysis** |
| Waste | Presence (Yes vs No) | Lithium mean concentration | Beta | -0.464 (-0.919, -0.01) | 0.045 | Main |
| Waste | Shipped tons (log1p) | Lithium mean concentration | Beta | -1.033 (-1.981, -0.085) | 0.033 | Main |
| Waste | Presence (Yes vs No) | Summed PFAS concentration | Beta | -0.051 (-0.175, 0.073) | 0.422 | Main |
| Waste | Shipped tons (log1p) | Summed PFAS concentration | Beta | 0.259 (0, 0.518) | 0.05 | Main |
| Waste | Shipped tons (log1p) | Detected PFAS analyte count | IRR | 1.25 (1, 1.54) | 0.045 | Main |
| Facility | Presence (Yes vs No) | Lithium mean concentration | Beta | -0.491 (-0.786, -0.196) | 0.001 | Main |
| Facility | Facility count (log1p) | Lithium mean concentration | Beta | -0.497 (-0.83, -0.164) | 0.003 | Main |
| Facility | Presence (Yes vs No) | Summed PFAS concentration | Beta | 0.065 (-0.016, 0.146) | 0.114 | Main |
| Facility | Facility count (log1p) | Summed PFAS concentration | Beta | 0.063 (-0.028, 0.154) | 0.176 | Main |
| Facility | Facility count (log1p) | Detected PFAS analyte count | IRR | 1.06 (0.96, 1.17) | 0.225 | Main |
| Waste | Presence (Yes vs No) | Lithium mean concentration | Beta | -0.41 (-0.867, 0.046) | 0.078 | Sensitivity |
| Waste | Shipped tons (log1p) | Lithium mean concentration | Beta | -0.929 (-1.88, 0.021) | 0.055 | Sensitivity |
| Waste | Presence (Yes vs No) | Summed PFAS concentration | Beta | -0.059 (-0.183, 0.066) | 0.358 | Sensitivity |
| Waste | Shipped tons (log1p) | Summed PFAS concentration | Beta | 0.247 (-0.013, 0.507) | 0.063 | Sensitivity |
| Waste | Shipped tons (log1p) | Detected PFAS analyte count | IRR | 1.23 (0.99, 1.53) | 0.059 | Sensitivity |
| Facility | Presence (Yes vs No) | Lithium mean concentration | Beta | -0.472 (-0.768, -0.176) | 0.002 | Sensitivity |
| Facility | Facility count (log1p) | Lithium mean concentration | Beta | -0.476 (-0.81, -0.142) | 0.005 | Sensitivity |
| Facility | Presence (Yes vs No) | Summed PFAS concentration | Beta | 0.065 (-0.016, 0.146) | 0.117 | Sensitivity |
| Facility | Facility count (log1p) | Summed PFAS concentration | Beta | 0.063 (-0.029, 0.154) | 0.18 | Sensitivity |
| Facility | Facility count (log1p) | Detected PFAS analyte count | IRR | 1.07 (0.96, 1.18) | 0.212 | Sensitivity |

| Supplemental Table 3. Regional sample sizes and LiB exposure indicators | | | | |
| --- | --- | --- | --- | --- |
| Region | Total ZIPs | Waste-active ZIPs, n (%) | ZIPs with ≥1 LiB facility, n (%) | Number of LiB facilities |
| Northeast | 3080 | 32 (1.0%) | 97 (3.1%) | 134 |
| Southeast | 5492 | 59 (1.1%) | 154 (2.8%) | 203 |
| Midwest | 4806 | 61 (1.3%) | 144 (3.0%) | 225 |
| Southwest | 1892 | 29 (1.5%) | 74 (3.9%) | 92 |
| West | 2419 | 68 (2.8%) | 162 (6.7%) | 249 |

| Supplemental Table 4. Full summary statistics for the US region stratified analysis. | | | | | |
| --- | --- | --- | --- | --- | --- |
| Region | **Exposure** | **Outcome** | **N ZIPs** | **Beta (95% CI)** | **P value** |
| Northeast | Waste presence | Lithium mean concentration | 3080 | -0.041 (-1.257, 1.175) | 0.947 |
| Northeast | Waste shipped tons | Lithium mean concentration | 3080 | -5.371 (-13.461, 2.718) | 0.193 |
| Northeast | Waste presence | Summed PFAS concentration | 3080 | -0.165 (-0.541, 0.21) | 0.389 |
| Northeast | Waste shipped tons | Summed PFAS concentration | 3080 | -0.406 (-2.906, 2.093) | 0.75 |
| Northeast | Facility presence | Lithium mean concentration | 3080 | -0.088 (-0.802, 0.626) | 0.808 |
| Northeast | Facility count | Lithium mean concentration | 3080 | -0.084 (-0.905, 0.737) | 0.841 |
| Northeast | Facility presence | Summed PFAS concentration | 3080 | 0.179 (-0.041, 0.4) | 0.111 |
| Northeast | Facility count | Summed PFAS concentration | 3080 | 0.208 (-0.046, 0.461) | 0.108 |
| Southeast | Waste presence | Lithium mean concentration | 5492 | -0.601 (-1.526, 0.324) | 0.203 |
| Southeast | Waste shipped tons | Lithium mean concentration | 5492 | 0.145 (-9.408, 9.697) | 0.976 |
| Southeast | Waste presence | Summed PFAS concentration | 5492 | -0.167 (-0.464, 0.13) | 0.27 |
| Southeast | Waste shipped tons | Summed PFAS concentration | 5492 | 1.765 (-1.303, 4.833) | 0.259 |
| Southeast | Facility presence | Lithium mean concentration | 5492 | -0.71 (-1.292, -0.128) | 0.017 |
| Southeast | Facility count | Lithium mean concentration | 5492 | -0.756 (-1.445, -0.066) | 0.032 |
| Southeast | Facility presence | Summed PFAS concentration | 5492 | 0.139 (-0.048, 0.326) | 0.146 |
| Southeast | Facility count | Summed PFAS concentration | 5492 | 0.127 (-0.094, 0.349) | 0.26 |
| Midwest | Waste presence | Lithium mean concentration | 4806 | -0.247 (-1.43, 0.936) | 0.682 |
| Midwest | Waste shipped tons | Lithium mean concentration | 4806 | -1.561 (-3.304, 0.182) | 0.079 |
| Midwest | Waste presence | Summed PFAS concentration | 4806 | 0.159 (-0.071, 0.39) | 0.175 |
| Midwest | Waste shipped tons | Summed PFAS concentration | 4806 | 0.372 (0.033, 0.712) | 0.032 |
| Midwest | Facility presence | Lithium mean concentration | 4806 | -1.871 (-2.658, -1.083) | <0.001 |
| Midwest | Facility count | Lithium mean concentration | 4806 | -1.923 (-2.767, -1.079) | <0.001 |
| Midwest | Facility presence | Summed PFAS concentration | 4806 | 0.052 (-0.102, 0.205) | 0.512 |
| Midwest | Facility count | Summed PFAS concentration | 4806 | 0.034 (-0.131, 0.198) | 0.69 |
| Southwest | Waste presence | Lithium mean concentration | 1892 | -0.61 (-2.122, 0.903) | 0.43 |
| Southwest | Waste shipped tons | Lithium mean concentration | 1892 | -3.155 (-7.147, 0.836) | 0.121 |
| Southwest | Waste presence | Summed PFAS concentration | 1892 | -0.229 (-0.625, 0.168) | 0.259 |
| Southwest | Waste shipped tons | Summed PFAS concentration | 1892 | 0.095 (-0.953, 1.143) | 0.859 |
| Southwest | Facility presence | Lithium mean concentration | 1892 | -0.583 (-1.548, 0.382) | 0.237 |
| Southwest | Facility count | Lithium mean concentration | 1892 | -0.478 (-1.666, 0.71) | 0.43 |
| Southwest | Facility presence | Summed PFAS concentration | 1892 | 0.169 (-0.084, 0.422) | 0.191 |
| Southwest | Facility count | Summed PFAS concentration | 1892 | 0.131 (-0.18, 0.443) | 0.409 |
| West | Waste presence | Lithium mean concentration | 2419 | -0.305 (-1.395, 0.784) | 0.583 |
| West | Waste shipped tons | Lithium mean concentration | 2419 | -0.681 (-2.473, 1.112) | 0.457 |
| West | Waste presence | Summed PFAS concentration | 2419 | -0.085 (-0.279, 0.108) | 0.388 |
| West | Waste shipped tons | Summed PFAS concentration | 2419 | 0.262 (-0.057, 0.581) | 0.108 |
| West | Facility presence | Lithium mean concentration | 2419 | 0.174 (-0.556, 0.904) | 0.641 |
| West | Facility count | Lithium mean concentration | 2419 | 0.197 (-0.59, 0.985) | 0.623 |
| West | Facility presence | Summed PFAS concentration | 2419 | -0.015 (-0.145, 0.115) | 0.821 |
| West | Facility count | Summed PFAS concentration | 2419 | 0.023 (-0.117, 0.163) | 0.749 |

| Supplemental Table 5. Spatial Sensitivity analyses. | | | | | | | | | | | | | | |
| --- | --- | --- | --- | --- | --- | --- | --- | --- | --- | --- | --- | --- | --- | --- |
| Exposure | Outcome | OLS beta | OLS lower 95% CI | OLS upper 95% CI | OLS P value | Residual Moran I | Residual Moran P value | Spatial beta | Spatial lower 95% CI | Spatial upper 95% CI | Spatial P value | Spatial approximate percent difference | Lambda | Lambda P value |
| Presence (Yes vs No) | Lithium mean concentration | -0.46 | -0.92 | -0.01 | 0.05 | 0.54 | 0.00 | -0.20 | -0.51 | 0.10 | 0.19 | -18.27 | 0.82 | 0.00 |
| Shipped tons (log1p) | Lithium mean concentration | -1.03 | -1.98 | -0.08 | 0.03 | 0.54 | 0.00 | -0.14 | -0.76 | 0.49 | 0.67 | -9.03 | 0.82 | 0.00 |
| Presence (Yes vs No) | Summed PFAS concentration | -0.05 | -0.18 | 0.07 | 0.42 | 0.57 | 0.00 | -0.04 | -0.12 | 0.04 | 0.37 | -3.64 | 0.82 | 0.00 |
| Shipped tons (log1p) | Summed PFAS concentration | 0.26 | 0.00 | 0.52 | 0.05 | 0.57 | 0.00 | 0.06 | -0.11 | 0.22 | 0.50 | 4.05 | 0.82 | 0.00 |
| Presence (Yes vs No) | Lithium mean concentration | -0.49 | -0.79 | -0.20 | 0.00 | 0.54 | 0.00 | -0.14 | -0.34 | 0.06 | 0.17 | -12.92 | 0.82 | 0.00 |
| Facility count (log1p) | Lithium mean concentration | -0.50 | -0.83 | -0.16 | 0.00 | 0.54 | 0.00 | -0.10 | -0.32 | 0.13 | 0.40 | -6.42 | 0.82 | 0.00 |
| Presence (Yes vs No) | Summed PFAS concentration | 0.06 | -0.02 | 0.15 | 0.11 | 0.57 | 0.00 | 0.07 | 0.01 | 0.12 | 0.01 | 6.97 | 0.82 | 0.00 |
| Facility count (log1p) | Summed PFAS concentration | 0.06 | -0.03 | 0.15 | 0.18 | 0.57 | 0.00 | 0.09 | 0.03 | 0.15 | 0.00 | 6.15 | 0.82 | 0.00 |
